# An observational study in psychiatric acute patients admitted to General Hospital Psychiatric Wards in Italy

**DOI:** 10.1186/1744-859X-6-2

**Published:** 2007-01-27

**Authors:** Andrea Ballerini, Roberto Boccalon, Giancarlo Boncompagni, Massimo Casacchia, Francesco Margari, Lina Minervini, Roberto Righi, Federico Russo, Andrea Salteri

**Affiliations:** 1 S. Maria Nuova Hospital, Florence, Italy; 2 Sant' Anna Hospital, Ferrara, Italy; 3 S.Orsola Malpighi Hospital, Bologna, Italy; 4 San Salvatore Hospital, L'Aquila, Italy; 5 Policlinico Consorziale Hospital, Bari, Italy; 6 Azienda USL 16 Hospital, Padua, Italy; 7 Hospital of Adria, Rovigo, Italy; 8 Nuovo Regina Margherita Hospital, Rome, Italy; 9 Vimercate Civil Hospital, Milan, Italy

## Abstract

**Objectives:**

this Italian observational study was aimed at collecting data of psychiatric patients with acute episodes entering General Hospital Psychiatric Wards (GHPWs). Information was focused on diagnosis (DSM-IV), reasons of hospitalisation, prescribed treatment, outcome of aggressive episodes, evolution of the acute episode.

**Methods:**

assessments were performed at admission and discharge. Used psychometric scales were the Brief Psychiatric Rating Scale (BPRS), the Modified Overt Aggression Scale (MOAS) and the Nurses' Observation Scale for Inpatient Evaluation (NOSIE-30).

**Results:**

864 adult patients were enrolled in 15 GHPWs: 728 (320 M; mean age 43.6 yrs) completed both admission and discharge visits. A severe psychotic episode with (19.1%) or without (47.7%) aggressive behaviour was the main reason of admission. Schizophrenia (42.8% at admission and 40.1% at discharge) and depression (12.9% at admission and 14.7% at discharge) were the predominant diagnoses. The mean hospital stay was 12 days. The mean (± SD) total score of MOAS at admission, day 7 and discharge was, respectively, 2.53 ± 5.1, 0.38 ± 2.2, and 0.21 ± 1.5. Forty-four (6.0%) patients had episodes of aggressiveness at admission and 8 (1.7%) at day 7. A progressive improvement in each domain/item vs. admission was observed for MOAS and BPRS, while NOSIE-30 did not change from day 4 onwards.

The number of patients with al least one psychotic drug taken at admission, in the first 7 days of hospitalisation, and prescribed at discharge, was, respectively: 472 (64.8%), 686 (94.2%) and 676 (92.9%). The respective most frequently psychotic drugs were: BDZs (60.6%, 85.7%, 69.5%), typical anti-psychotics (48.3%, 57.0%, 49.6%), atypical anti-psychotics (35.6%, 41.8%, 39.8%) and antidepressants (40.9%, 48.8%, 43.2%). Rates of patients with one, two or > 2 psychotic drugs taken at admission and day 7, and prescribed at discharge, were, respectively: 24.8%, 8.2% and 13.5% in mono-therapy; 22.0%, 20.6% and 26.6% with two drugs, and 53.2%, 57.8% and 59.0% with > two drugs. Benzodiazepines were the most common drugs both at admission (60.0%) and during hospitalisation (85.7%), and 69.5% were prescribed at discharge.

**Conclusion:**

patients with psychiatric diseases in acute phase experienced a satisfactory outcome following intensified therapeutic interventions during hospitalisation.

## Background

Despite an increasing amount of studies on the epidemiology of acute mental disorders and the availability of recently introduced pharmacological interventions in the management of such conditions, only few reports provide detailed information on the characteristics of psychiatric patients and treatments received both in the hospital setting and as routine clinical practice [1].

In Italy, a law issued in 1978 stated that all admission of psychiatric patients had to take place in the General Hospital Psychiatric Wards (GHPWs), thus prohibiting the admission to Psychiatric Hospitals. From then on, very few epidemiological studies have been carried out on inpatient population with psychiatric disorders. Furthermore, most of reports refer to studies performed in local settings [2-4], which may differ between them in terms of methods of admission, patients' demographics and socio-cultural background, and interventions.

GHPWs are psychiatric centres for acute patients with any psychiatric-related illness, and are located in General Hospitals. Patients remain in GHPWs only during the acute phase. At discharge, they usually receive therapeutic prescriptions and are no more followed by GHPWs structures, but they are followed by territorial services, which are not part of General Hospitals. Little is known about therapies used in GHPWs.

Recent National epidemiological studies have also shown that the rates of first admission diagnosis in Italy may differ from other Western countries [5], and that therapeutic interventions may depend more on physicians' experience, common sense and other cultural parameters rather than on a more rational approach on drug use [6].

Daily living conditions of Italian psychiatric patients, such as living alone or with relatives, also differ from that of other countries [7]. Another confounding factor may consist in the evidence that a significant proportion of subjects attending GHPWs are 'self-referred' patients and less than half admissions are referred by a qualified psychiatrist [8]. Furthermore, in Italy drug dependent patients are managed by different medical services independent both by hospital and territorial services.

Therefore, different Psychiatric Departments' organisation and the availability of newly introduced drugs (e.g. atypical antipsychotic) may cause marked differences among countries, and even among different regions in the same country, both in terms of diagnosis at admission and discharge, and in terms of therapeutic intervention over time.

Based on the above considerations, to better understand role and function of GHPWs uniformly distributed across the National territory, the EPICA ('Gruppo di Studio Epidemiologia in Psichiatria Casi Acuti') study group was aimed at collecting data of adult psychiatric inpatients entering the study with different diagnosis. Assessment of effects of interventional measures by using appropriate and validated psychometric scales was the main objective of this observational study.

EPICA was a pilot study for the preparation of a more comprehensive study on GHPWs in Italy, the PERSEO (Psychiatric EmeRgency Study and EpidemiOlogy) Study.

## Patients and methods

### Patients and diagnosis

Patients afferent to GHPWs from March 25^th ^2002 to July 26^th ^2002 were eligible for the study. Fifteen sites took part in the study. Patients previously enrolled in this study and newly admitted to GHPWs were excluded from participation; however, any new admission was recorded in the case report form.

Descriptive epidemiology included the analysis of diagnoses distribution according to DSM-IV and ICD-9, and the evaluation of social and demographic profile of patient population, the reason of hospitalisation and the interventional procedures. Clinical epidemiology was based on the assessment of prevalence of aggressive episodes at admission and their incidence in the observational period; the evolution of the acute psychotic episode (diagnosis, treatment and outcome) was also evaluated. The outcome of the acute episode was evaluated in patients with one of the following group of diagnoses: schizophrenia, depression, nevrotic disturbance, substance abuse, psychorganic psychoses, mania, undifferentiated, antisocial and non-antisocial personality disorder.

### Observational period

**Figure 1:**
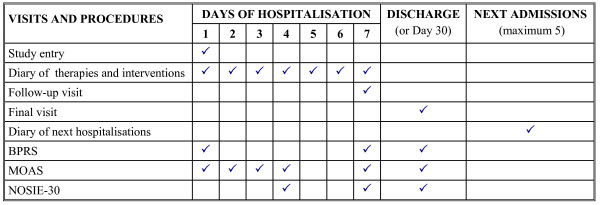
Study flow-chart and procedures.

The study design and procedures, including time of assessment, are summarized in Figure 1. The maximal observational length was 30 days; daily recording of interventions and outcome was performed in the first 7 days of hospitalisation. Visits at Psychiatric Wards were scheduled at study entry (day 1, admission), at follow-up (day 7) and at discharge (final visit). On day 30, observation was discontinued anyway and assessments of final visit were performed. In patients discharged prior to or at day 7, forms for final visit were to be completed, without any follow-up observation. A form for the next 5 hospitalisations following that of the present study was also to be completed.

### Psychometric scales

The following psychometric scales were used for assessment: BPRS (Brief Psychiatric Rating Scale), MOAS (Modified Overt Aggression Scale), and NOSIE-30 (Nurses' Observation Scale for Inpatient Evaluation).

BPRS is a validated and widely used psychometric scale. An Italian expanded 24-item version (BPRS version 4.0) was used in this study [9]. Severity of a total of 24 symptoms, grouped in 6 different domains, was evaluated using a 7-point rating scale ranging from 'not present' to 'extremely severe' to obtain an overall total score. High levels of inter-rate reliability between experienced (i.e. psychiatrists and psychologists) and inexperienced operators (i.e. medical and psychosocial rehabilitation students) were shown in previous trials [10]. BPRS version 4.0 was administered at admission, at day 7 and at discharge (or day 30) and forms were completed by physicians following a patient's interview.

The MOAS scale [11] records the forms of aggression and their severity; it is constituted by 4 subscales based on increasing severity: verbal aggression, aggression towards properties, self-aggression, and physical aggression towards people. Each subscale includes 5 items scored 0-4; the total score is obtained by multiplying scores of each subscale by their specific 'weight' (1, 2, 3 and 4, respectively), then adding the 4 obtained values. Subjects with aggressive behaviours are defined as those having a total score > 0 in the observational period. MOAS was completed by non-medical healthcare personnel to assess outcome or onset of aggressive episodes; it was administered at admission, at day 2, 3, 4 and 7, and at discharge (or day 30).

The NOSIE-30 [12] was used to assess frequency of 30 behaviours in hospitalised patients, ranging from 'never' to 'always'. The 30 items are divided in 7 different domains: social competence, social interest, personal neatness, irritability, manifest psychosis, retardation and depression. The first 3 domains reflect positive behavioural dimensions (Total Positive Factors), and the other 4 are indicators of negative behaviours (Total Negative Factors). The Total Patient Asset score was obtained by the sum of the positive factors minus the sum of negative factors and adding 150 as a normalisation factor.

### Study organisation

One Local Study Coordinator (LSC) was identified in each participating site. LSC was responsible for study conduction and study material distribution; he/she also identified and properly trained the Clinical Investigators (CI) and Raters, and ensured their correct application of study procedures. CIs were responsible for patients' selection and enrolment, BPRS administration according to protocol, completion of case report forms, and contact with Data Management centre (including quality controls). Raters were charged of MOAS and NOSIE-30 administration and correctness.

### Data analysis

Data Management, quality control and Statistics were performed by Runtimes srl, Modena (Italy). Results of parametric variables were presented as means ± standard deviation and range, while results of categorical variables were presented as number and proportions. Evaluable patients were those completing both the admission and the discharge visits.

## Results

### Patients' general characteristics

The general characteristics of patients' populations are summarised in Table 1. A total of 864 patients were enrolled in 15 GHPWs and 728 of them (320 males and 408 females) resulted to be evaluable (i.e. had both admission and discharge visits). The mean age (± standard deviation) was 43.6 ± 14.8 years (range 16-99) in the total population, and was slightly higher in females than in males. Most of patients were included in the age ranges of 26-34 (164 patients), 35-44 (177 patients), 45-54 (131 patients) and 55-64 (108 patients) years. With regards to life habits, most of patients were non-alcohol users (402, 55.2%) and non-drug users (602, 82.7%), while more smokers (385, 52.9%) than non-smokers (291, 40.0%) took part in the study.

**Table 1:** Patients' characteristics

Patient disposition *(number and percentages)*	
No. of enrolled patients	864
No. of patients with admission visit	728 (84.3% of enrolled)
No. of patients with follow-up visit	428 (58.8% of evaluable)
No. of patients with discharge visit	728 (84.3% of enrolled)
Sex *(number and percentages)*	
Males	320 (44.0)
Females	408 (56.0)
Age, years *(mean ± SD, range in brackets)*	
Total population	43.56 ± 14.8 (16-99)
Males	41.74 ± 15.0 (17-99)
Females	44.99 ± 14.5 (16-99)
Age ranges *(number and percentages in brackets)*	
≤25 years	68 (9.3)
26-34 years	164 (22.5)
35-44 years	177 (24.3)
45-54 years	131 (18.0)
55-64 years	108 (14.8)
65-74 years	58 (14.8)
≥75 years	14 (1.9)
NR	8 (1.1)
Weight, kg *(mean ± SD, range in brackets)*	71.2 ± 17.2 (32-155)
Height, cm *(mean ± SD, range in brackets)*	167.5 ± 9.2 (140-195)
Alcohol users *(number and percentages in brackets)*	
No	402 (55.2)
Yes, without excess	149 (20.5)
Yes, with excess	135 (18.5)
NR	42 (5.8)
Smoke habits *(number and percentages in brackets)*	
Non-smokers	291 (40.0)
Smokers	385 (52.9)
Ex-smokers	16 (2.2)
NR	36 (4.9)
Substance abuse *(number and percentages in brackets)*	
No	602 (82.7)
Yes	47 (6.5)
Ex-users	30 (4.1)
NR	49 (6.7)

NR: Not recorded

The main reason of admission was a severe psychotic episode with (139 patients, 19.1%) or without (347, 47.7%) aggressive behaviour; less frequent reasons included moderate psychoses with unavailability of any caregiver (108, 14.8%) and Axis I disorders with alcohol abuse (56, 7.7%).

The most frequent patients' referrals were the Hospital emergency department (245, 33.7%), a mental-care centre (119, 16.3%) and self-referral (103, 14.1%); 429 patients (58.9%) had a known diagnosis at admission. The mean number of hospitalisations in the previous 12 months was 1.12 ± 2.51.

### Hospitalisation and diagnosis

The mean length of hospitalisation was 12.0 ± 10.2 days (range 1-92); 24.7% of patients stayed in GHPWs for more than 15 days, 17.9% for 11 to 15 days and 16.9% for 8 to 10 days, while lower rates of patients had a shorter stay.

**Figure 2:**
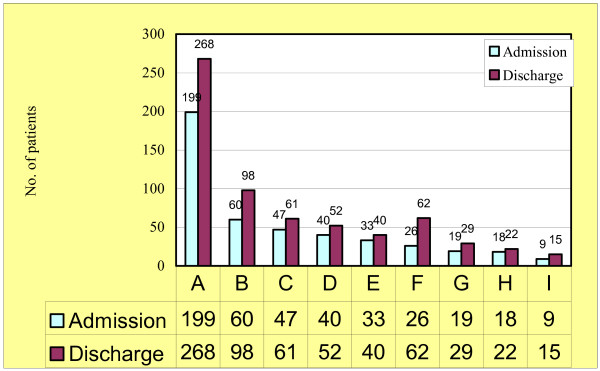
Groups of diagnosis at admission and discharge. A = Schizophrenia; B = Depression; C = Undifferentiated Personality Disorder; D = Mania; E = Non-antisocial Personality Disorder; F = Nevrotic Disturbance; G = Psychorganic Psychosis; H = Substance Abuse; I = Antisocial Personality Disorder.

Primary diagnoses at admission and discharge are presented in Figure 2. The most frequent groups of diagnoses (according to ICD-9) at admission were schizophrenia (199 patients, 42.8%), depression (60 patients, 12.9%) and undifferentiated personality disorder (47, 10.1%). Less frequent diagnoses included mania (40, 8.6%), non-antisocial personality disorder (33, 7.1%), nevrotic disturbance (26, 5.6%), psychorganic psychoses (19, 4.1%), substance abuse (18, 3.9%) and antisocial personality disorder (9, 1.9%). Diagnosis at discharge were schizophrenia (268 patients, 40.1%), depression (98 patients, 14.7%), nevrotic disturbance (62, 9.3%), undifferentiated personality disorder (61, 9.1%), mania (52, 7.8%), non-antisocial personality disorder (40, 6.0%), psychorganic psychoses (29, 4.3%), substance abuse (22, 3.3%) and antisocial personality disorder (15, 2.2%). Diagnosis at admission was not available in 263 patients (36.1%), while 59 patients (8.1%) were not diagnosed at discharge.

A total of 59 patients (8.1%) had at least one further hospitalisation after discharge: the mean number of further admissions was 1.39 ± 0.81 and mean duration was 10.68 ± 8.72 days; main reasons were a severe psychotic episode with (9 patients, 11.0%) or without (40, 48.8%) aggressiveness.

### Psychometric scales

The number of patients with at least one episode of aggressiveness was 44 (6.0% of hospitalised) at admission and progressively declined over time: they were 24 (3.4%) at day 2, 13 (1.9%) at day 3, 9 (1.4%) at day 4, and 8 (1.7%) at day 7.

**Figure 3:**
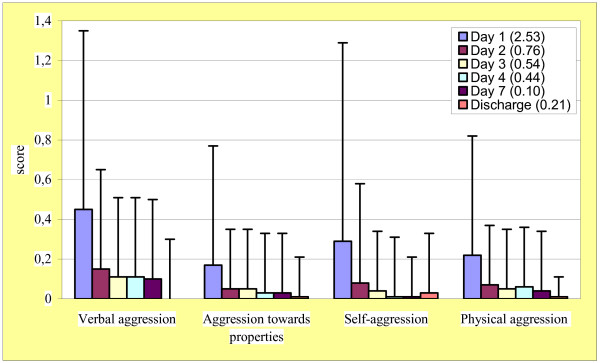
Results of MOAS subscores (values are means, standard deviations in bars); total scores in brackets.

Results of MOAS are presented in Figure 3. A marked and progressive decrease of mean scores from admission to discharge was observed in total score and in each domain. Total score was 2.53 ± 5.1 at admission, 0.38 ± 2.2 at day 7 and 0.21 ± 1.5 at discharge; changes of single domains (verbal aggression, aggression towards properties, self-aggression, and physical aggression towards people) were consistent with those of total score.

**Figure 4:**
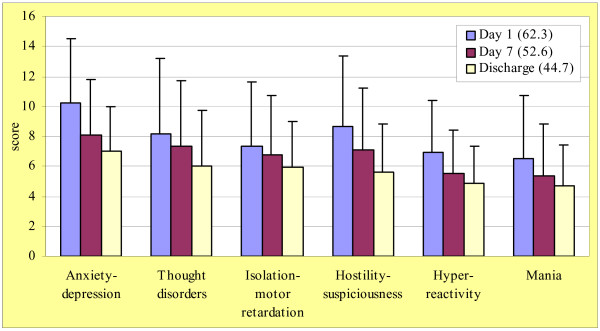
Results of BPRS subscores (values are means, standard deviations in bars); total scores in brackets.

Figure 4 shows results of BPRS version 4.0. Total score was 62.3 ± 21.2 at admission (day 1), 52.6 ± 20.7 at day 7 and 44.7 ± 17.3 at discharge. A progressive decrease over time of values recorded at study entry was also observed in each domain (anxiety-depression, thought disorders, isolation-motor retardation, hostility-suspiciousness, hyper-reactivity, and mania) as well as in each of the 24 single items. Improvements at discharge were observed in each group of diagnosis (data not shown) and were of similar extent in all investigated domains.

Results of NOSIE-30 recorded at day 4, day 7 and discharge are presented in Table 2. No clinically relevant changes were observed in any of the investigated domains from day 4 to discharge. Among 'positive' domains, a small increase was observed in social interest, compared with a small decline in social competence; among 'negative' domains, all of them (irritability, manifest psychosis, retardation and depression) showed small improvements at discharge. Mean values of total patient's asset also did not change from day 4 to discharge and no changes were also observed grouping patients by diagnosis.

**Table 2:** Results of NOSIE-30 (means ± standard deviations).

DOMAINS	**Day 4**	**Day 7**	**Discharge**
Social competence	9.0 ± 3.8	8.7 ± 3.8	8.0 ± 3.3
Social interest	10.9 ± 3.6	11.2 ± 3.7	11.8 ± 3.8
Personal neatness	9.4 ± 1.9	9.4 ± 1.9	9.4 ± 1.9
Irritability	10.9 ± 4.9	10.2 ± 4.5	10.2 ± 4.3
Manifest psychosis	5.7 ± 2.5	5.7 ± 2.6	5.2 ± 2.0
Retardation	6.4 ± 2.8	5.9 ± 2.6	5.4 ± 2.3
Depression	5.1 ± 2.3	4.8 ± 1.9	4.6 ± 1.9

TOTAL PATIENT ASSET*	151 ± 8.0	153 ± 7.6	154 ± 7.8

*Total Patient Asset score was obtained by the sum of the positive factors minus the sum of negative factors and adding 150 as a normalisation factor

### Therapeutical interventions

Table 3 shows the number of patients with at least one psychotic drug taken at study entry, during hospitalisation (day 1, day 4 and day 7) and at discharge.

**Table 3:** Number of patients (percentage in brackets) with least one psychotic drug taken at study entry, during hospitalisation and at discharge.

	**Study entry**	**Day 1**	**Day 4**	**Day 7**	**Discharge**
Patients taking at least one drug	472 (64.8)	661 (90.8)	601 (94.9)	461 (95.2)	676 (92.9)
Monotherapy	117 (24.8)	87 (13.2)	43 (7.2)	38 (8.2)	91 (13.5)
Combination with 2 drugs	104 (22.0)	192 (29.0)	141 (23.5)	95 (20.6)	180 (26.6)
Combination with 3 drugs	128 (27.1)	191 (28.9)	180 (30.0)	132 (28.6)	196 (29.0)
Combination with 4 drugs	89 (18.9)	128 (19.4)	140 (23.3)	110 (23.9)	142 (21.0)
Combination with > 4 drugs	34 (7.2)	50 (7.6)	65 (10.8)	52 (11.3)	61 (9.0)
Mean number of drugs (± SD)	2.6 ± 1.3	2.9 ± 1.2	3.2 ± 1.3	3.3 ± 1.4	2.9 ± 1.2

A number of 472 (64.8%) patients were previously taking at least one drug at study entry, and this amount increased from day 1 (90.8%) to day 7 (95.2%); 686 (94.2%) patients received drug therapy during hospitalisation and 676 (92.9%) had at least one psychotropic drug prescribed at discharge. Monotherapy was administered in 117 patients (24.8%) prior to admission, decreased during hospitalisation (13.2% at day 1, 7.2% at day 4 and 8.2% at day 7), and was prescribed in 91 patients (13.5%) at discharge, in favour of a more intensive treatment that included combined therapies with increased frequency. Rates of patients with two psychotic drugs taken at admission and day 7, and prescribed at discharge, were, respectively, 22.0%, 20.6% and 26.6%; the corresponding figures of polytherapy with more than 2 drugs were 53.2%, 57.8% and 59.0%.

As a consequence, the mean number of psychotic drugs taken simultaneously was 2.6 ± 1.3 prior to admission and increased to 2.9 ± 1.2 at day 1, 3.2 ± 1.3 at day 4, and 3.3 ± 1.4 at day 7; the mean number at discharge was 2.9 ± 1.2.

The most frequently psychotic drug classes taken at study entry, and prescribed in the first 7 days of hospitalisation and at discharge, are presented in Table 4. Benzodiazepines were the most frequently used drugs at study entry (60.0% of patients); their administration was intensified during hospitalisation (85.7%) and were prescribed in 69.5% of patients at discharge. Rates of inpatients treated with other drugs also increased: typical antipsychotics were taken at study entry and during hospitalisation in 48.3% and 57.0% of patients, respectively, and were also prescribed at discharge in 49.6%; the corresponding figures were 35.6%, 41.8% and 39.8% for atypical antipsychotic drugs, 40.9%, 48.8% and 43.2% for antidepressants, and 23.9%, 27.1% and 29.1% for mood stabilizers. Less frequently used other drugs included antidotes for drug of abuse.

**Table 4:** Most frequently psychoactive drug classes taken at study entry, in the first 7 days of hospitalisation and prescribed at discharge (numbers are patients; percentages in brackets refer to total amount of treated/prescribed patients).

DRUG CLASSES	**Study entry**	**During first 7 days**	**Prescribed at discharge**
Benzodiazepines	286 (60.0)	588 (85.7)	470 (69.5)
Typical anti-psychotic drugs	228 (48.3)	391 (57.0)	335 (49.6)
Atypical anti-psychotic drugs	168 (35.6)	287 (41.8)	269 (39.8)
Antidepressants	193 (40.9)	335 (48.8)	292 (43.2)
Mood stabilisers	113 (23.9)	186 (27.1)	197 (29.1)
Other drugs	86 (18.2)	165 (24.1)	122 (18.0)

No. of patients treated at study entry: 472; No. of patients treated during the first 7 days of hospitalisation: 686; No. of patients prescribed at discharge: 676

In the overall population, the most frequently used drugs prior to study entry were haloperidol (25.6% of patients), delorazepam (20.1%) and lorazepam (19.5%); predominant drugs during hospitalisation were delorazepam (35.9%), lorazepam (26.7%) and haloperidol (25.4%), while the most frequently prescribed drug at hospital discharge were haloperidol (26.3%), delorazepam (25.7%) and olanzapine (21.2%). Predominant combined therapies administered in inpatients were benzodiazepines-antidepressants (15.6% of patients), atypical drugs-benzodiazepines (14.9%) and benzodiazepines-antidepressants-atypical drugs (9.5%).

## Discussion

The main results of this observational study conducted in 15 GHPWs distributed across the whole Italian territory showed that patients with psychiatric diseases in acute phase benefited from intensified therapeutical interventions during hospitalisation.

Schizophrenic disorders were the most frequent diagnosis recorded at entry and accounted for approximately half of diagnoses. Diagnosis at discharge showed that schizophrenia, depression and nevrotic disorders were all diagnosed in an higher proportion of patients compared to admission. Changes of diagnosis and required treatment for the acute episode led to pharmaceutical intervention during hospitalisation and drug prescription at discharge which was markedly different from that recorded at admission. However, it can be considered that a significant proportion of patients (more than 40% of total evaluable sample) had a missing diagnosis at entry, while only approximately 15% of participating subjects were not diagnosed at discharge.

Treatment of inpatients included combined therapies in 84.4% of cases at day 7; a therapy with 2 or more drugs was prescribed in 85.6% of patients at discharge, compared to 75.2% at admission. Treatment or prescription of combined therapy with 3 or more psychotic drugs also increased during hospitalisation and at discharge, respectively. With regards to prescription at discharge, BDZs resulted to be predominant and rates of prescribed patients increased compared with users at admission; rates of patients prescribed at discharge with drug of other classes also increased vs. pre-hospitalisation.

Intensified interventions led to a satisfactory outcome of the psychotic acute episode. Aggressiveness, as measured using MOAS, progressively decreased during hospitalisation and at discharge compared to admission, as well as scores of each domain tended to zero (i.e. absence of aggressiveness) at discharge. Results of BPRS also showed a progressive decrease over time both of total score and single domains (and items). Changes of MOAS and BPRS were observed irrespective of the diagnosis.

Results of NOSIE-30 did not show evidence of changes of behaviours from day 4 to discharge: according with previous findings in schizophrenic patients [13], it is likely that longer periods of observation are required to detect reliable changes. Also, assessments started from day 4 and, therefore, potential early changes due to interventions were not measured.

The consumption of psychoactive drugs in Italy (particularly antidepressants) is known to be relatively lower compared to that reported in other countries [14]; this might be due to cultural or economical factors. However, the prescription of such drugs is rarely consistent with standards of treatment recommended by Health authority [15]. A recent survey on the treatment of schizophrenia in Italy [16] has also shown that polypharmacy and neuroleptics were administered outside the recommended dose ranges and durations, and that treatment regimens of the various drug classes diverged on the basis of patients' age, population density and geographical area. For example, patients treated with atypical antipsychotic drugs are mainly younger than those receiving other drug classes and polytherapy is more frequently prescribed in patients receiving typical antipsychotic drugs [16]; furthermore, prescription of antidepressants in Southern Italy is lower than that in the rest of the country [17]. This study was designed to obtain an overall overview in terms of diagnosis and effects of therapy, regardless of location and trend of treatment in individual sites; however, results seem to confirm a sub optimal treatment of psychiatric outpatients. Diagnosis at entry was also missing in a relevant amount of patients, mainly because of the prioritisation to symptoms relief, postponing diagnosis after patient was stabilised.

Onset of acute episodes might also bee due to a lack of compliance to prescribed drug regimens: it is well recognised that the administered dose in a domiciliary setting is often much less than prescribed or even omitted at all [18]. Therefore, treatment of inpatients is useful to avoid problems of misuse and to adopt therapies according with a more precise diagnosis.

The pattern of use of antipsychotic drugs is greatly changed in recent years, particularly after the introduction of atypical drugs, and emerging trends towards an intensified drug dosing and polytherapy have been described worldwide [19]. Findings of this study are consistent with this current trend in use of antipsychotics on inpatients basis.

The General Hospital Psychiatric Wards setting is the structure suitable for the treatment of acute psychiatric cases; more rational and intensified use of psychotropic drugs can be recommended to achieve a rapid and favourable response to therapy. Since antipsychotics were the most widely used drugs in acute hospitalization phase, the availability of newer formulations with faster onset of action and better safety profile offered by atypical drugs, which were not yet in use at the time of this study, will allow advances in pharmacological treatment.
